# Design and analysis of a highly sensitive SPR based PCF biosensor with double step dual peak shift sensitivity

**DOI:** 10.1016/j.heliyon.2023.e18782

**Published:** 2023-07-28

**Authors:** Mohammad Rakibul Islam, Md Moinul Islam Khan, Ahmad Jarif Yeasir, Fariha Mehjabin, Jannat Ara Mim, Jubair Alam Chowdhury, Tajuddin Ahmed Nahid, Mohibul Islam

**Affiliations:** Electrical and Electronic Engineering Department, Islamic University of Technology, Board Bazar, Gazipur-1704, Bangladesh

**Keywords:** RI sensor, Temperature sensor, Surface plasmon resonance, Wavelength sensitivity, Amplitude sensitivity, Double step dual peak shift sensitivity

## Abstract

This paper introduces a comprehensive study of a quad-cluster multi-functional Photonic Crystal Fiber (PCF) sensor where gold and Aluminum doped with zinc oxide (AZO) were used as plasmonic materials. A maximum Amplitude Sensitivity (AS) of 5336 RIU^−1^ and Wavelength Sensitivity (WS) of 40,500 nm/RIU in y pol was obtained incorporating Gold as plasmonic material. When AZO was included as the plasmonic material, AS of 3763 RIU^−1^ & WS of 9100 nm/RIU for y polarization were determined. The RI detecting range was increased from 1.32 to 1.43 to 1.19–1.42 after using AZO instead of Au that opens up a new horizon for detection. A novel detection technique, ‘Double Step Dual Peak Shift Sensitivity (DS-DPSS)’ was proposed in sensing temperature where highest sensitivity of 1.05 nm/^°^C having resolution of 0.095 °C for x pol. was achieved. Due to its diverse functionality, the suggested sensor represents a significant advancement in the detection of numerous analytes in biochemical applications.

## 1 Introduction

Plasmonic sensors are developed on the concept of surface plasmon resonance (SPR) phenomena, which occurs at the interface of metal-dielectric under certain special conditions [1]. When an electromagnetic (EM) wave with a p-polarization strikes the interface, it causes total internal reflection (TIR), which creates an evanescent field that excites electrons in the metal. So, surface plasmon polaritons (SPPs) are made when electrons at the surface of a metal move around together. The length or angle of the p-polarized wave varies when it resonates with the SPPs on the metal's surface. Because EM waves keep oscillating, their energy transfers to SPPs, where it generates surface plasmon waves (SPWs). They respond sensitively to surrounding RI. So, even a small change in RI medium is understood by looking at how the resonance angle or wavelength changes [2]. This phenomenon is used to detect different biological, chemical purposes, for environmental monitoring, food testing, salinity measurement [3,4] as well as detecting temperature [[5], [6], [7]]. Some analytes (such as toluene, polydimethylsiloxane, ethanol, benzene, chloroform) are sensitive to temperature. Thus, their RI changes with the environmental temperature. Whenever there is a fluctuation in the analyte's temperature, resonance wavelength shifts which allows the detection of the temperature. This unique property can be used for many critical applications, including biological and medical monitoring, acetone condensation, brain tumor treatment, industrial production, and so on [5,6,8,9].

Current SPR sensors mostly depend on either prism coupling or optical fibers [10]. Kretschmann configuration is commonly used for prism coupling coating plasmonic metal onto the base of a prism [11]. SPR only occurs in this setup when the incidence angle of the input wave is greater than the critical angle. To attain this precise angle, however, a few optical and mechanical components are employed, making the device hefty and expensive. Thus, it has become less feasible to fabricate and is inadequate for sensing remotely [12]. To subdue these limitations, PCF merged with SPR came into light. Photonic crystal fibers (PCF) have recently been used in many areas, like optical sensors [[13], [14], [15]], SPR sensors [16] and optical communication [[17], [18], [19], [20]]. It is better than fiber-based SPR sensors and usual prisms because of its various advantages, such as operation in single mood, notable nonlinearity, birefringence, and extremely low propagation loss [21]. Because of its flexible, strong, and small structural design, it can be used in optical devices and fiber lasers [22]. PCF sensors have opened the door to a new era in biological and chemical sensing owing to advances in manufacturing and sensing technologies. In contrast, these have been found to have limited sensitivity and substantial propagation loss in earlier studies. To achieve high sensitivity and propagation loss relatively low, researchers are currently focusing on modifying the PCF's geometrical features which include the diameter of air hole, pitch, number of holes in the cladding, fiber's length and lattice arrangement. Selecting plasmonic material for a PCF sensor is crucial, as it greatly affects the sensor's performance [23].

So far, numerous metals such as Au (Gold), Ag (Silver), Cu (Copper) and Al (Aluminum) have been used as plasmonic material to improve the sensitivity of the PCF sensor [24]. Unfortunately, such sensors being affected by several downsides resulted in unwanted features of these plasmonic materials. One such disadvantage is that instead of having a sharp resonance peak in the case of Ag, it has failed to grab attention because of its oxidation problem [1]. This drawback can be solved with another of graphene or TiO_2_ [25], but unfortunately, this extra layer causes fabrication complexity. Due to its high electron density and minimal damping loss, Aluminum has a great deal of potential as a plasmonic material, but it is also limited by oxidation [26]. Aluminum-doped Zinc-oxide (AZO) can be a potential candidate for plasmonic material as it has significantly lower loss than silver [27] and a sharp resonance peak. However, gold (Au) has been the most recommended plasmonic material [[28], [29], [30]] due to its desirable features like chemical inertness and doesn't suffer from oxidation [26]. On the other hand, gold doesn't stick well to silica, which makes it perform poorly. This obstacle can be fixed by adding a thin layer of TiO_2_.

Different kinds of structures have been designed and investigated by researchers to enhance sensitivity and reduce the fabrication difficulty, such as D-shaped, internal sensing, and sensing externally. For designs which involve sensing internally, air holes need to be coated with plasmonic material, and an analyte is injected into air holes. Fabrication is complicated by the internal metal coating, which drives up production costs, and use is difficult because it requires injecting analyte into specific air holes and extracting it to inject newer ones [30]. Nowadays, the D-shaped structure has grabbed the attention of researchers as the layer of metal can be easily polished on the flat surface [31]. I. Danlard et al. [32] mentioned using a dual-polarized quasi-D-shaped PCF for RI and temperature monitoring in 2021. For RI detection, maximal sensitivity of wavelength was found 5000 nm/RIU and maximal sensitivity of amplitude was 266.54 RIU^−1^ with resolution of 2 × 10^−5^ RIU and for temperature, maximum sensitivity is 3 nm/^°^C. Recently Farhan et al. [33] has proposed a D-shaped biosensor with maximum sensitivity of 47,260 nm/RIU. However, although D-shaped can be metal-coated more readily than internal sensing, a flat surface requires precise side refining.

Thus, till now, as the plasmonic matter deposits on the surface outside of the sensor, the most desirable configuration is that of a metal coating externally on the sensor. A recent anisotropic PCF design proposed by A.K Shakya et al. [34] shows a maximum WS of 20,000 nm/RIU and a maximum AS of 3167 RIU^−1^ with resolution of 5 × 10^−6^ RIU. In 2022, M.R Islam et al. [35] proposed a bi-cluster and double array-based biosensor with maximum amplitude sensitivity of 3807 RIU^−1^ with resolution of 2.63 × 10^−6^ RIU and maximum wavelength sensitivity of 80,500 nm/RIU with resolution of 1.24 × 10^−6^ RIU. In 2021, a SPR-based temperature sensor [36] with a sensitivity of 1.151 nm/^o^C from 20 °C to 70 °C and gold as plasmonic material was introduced. Farhan et al. [37] proposed a multi-channel sensor which will simultaneously detect three analytes with maximum sensitivity of 3292 nm/RIU (Channel-1), 6664 nm/RIU (Channel-2), and 10,243 nm/RIU (Channel-3).

In this manuscript, we proposed a multi-purpose PCF sensor based on SPR whose performance is investigated using two different plasmonic materials, gold and AZO, respectively. It has two types of circular air holes. A thin layer of TiO_2_ has been used to increase adhesion of the plasmonic material and thus enhance the sensing performance. An investigation has been performed on the proposed sensor's different parameters to maximize the wavelength and amplitude sensitivity, FOM, and temperature sensitivity using FEM of COMSOL. Along with the conventional method, a dual-step temperature verification method is introduced here which has not been studied earlier in any other work to the best of our knowledge.

## 2 Geometry and fabrication modelling

### 2.1 Proposed sensing system design

**Fig. 1:**
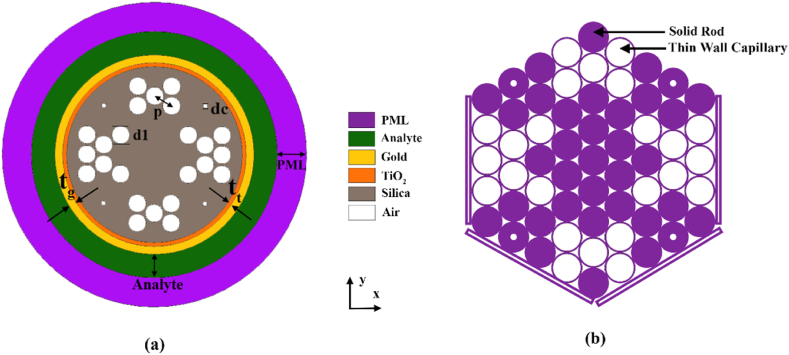
(a) Schematic of the suggested SPR-based biosensor showing its different components at cross-section; (b) Stackable PCF preform design.

The cross section consists of quad clusters of air holes (circular) in vertical and horizontal symmetric orientation as illustrated in Fig. 1 (a). The upper and lower clusters are made of air holes, making an X-shaped pattern all of which has a center-to-center distance, pitch p = 1.0 μm constant all over the cladding region. As shown in Fig. 1(b), the design includes two types of air holes: four corner air holes with a relatively small diameter d_c_ and cluster air holes with a standard diameter d_1_. To enhance the interaction between the evanescent field and the plasmonic gold layer during SPP mode, the smallest air holes are placed at four corners of the cladding region. Meanwhile, the air hole clusters on the right and left side are made π-shaped for proper confinement of propagated light in the solid core. The fiber is composed of many layers, beginning with the most outer layer of PML, indicated as t_PML_ in Fig. 1 (a), which measures 1.50 μm in thickness. This manufactured PML layer helps in transmitted energy absorption, and no EM wave refraction happens at the shiny gold surface. Behind PML layer lies the analyte layer t_a_, which the sensor detects. To optimize the EM field containment that happens in the core directed mode, the core center has been maintained rigid and clear of air openings. Solid core mode traps the light right into the middle of fiber producing the core confinement loss. Numerical computations imply the sample layer thickness as 1.20 μm which has a considerable impact on the sensitivity. The framework material of the sensor is fused silica (SiO_2_), & the sensing layer is a 20 nm gold-coated surface. Gold is preferred as plasmonic material for showing more chemical stability over other metals. Besides, broader shifts of resonance wavelengths also serve as a distinct property for gold. Chemical vapor deposition (CVD) can be utilized to obtain the gold covering [38]. In the cladding region, corner air holes having smaller diameter (d_c_ = 0.2 μm) than the rest (d_1_ = 0.90 μm), give rise to leakage. The evanescent field excites the electrons in the gold layer, causing SPR at the metal-dielectric interface. The precision has been increased by fabricating the elements with a finer grain size. For absolute precision of data analysis, both x-polarization & y-polarization were described. Finite Element Method is used to compute & study sensor function performance. The proposed PCF sensor is designed with a core of 2.6 μm diameter and cladding region of 6.4 μm diameter. Using a range of different optical parameters, their values have been determined to be as follows: *t*_*a*_ = 1.2 μm, *t*_*PML*_ = 1.5 μm, *t*_*t*_ = 10 nm, *t*_*g*_ = 20 nm, *p* (pitch) = 1.0 μm, *d*_*1*_ = 0.9 μm and *d*_*c*_ = 0.2 μm.

Sellmeier equation is used to determine RI of background material SiO_2_. The RI is obtained from the equation [39]:

(1) $$n_{si}^{2}=1+\frac{A_{1}\lambda^{2}}{\lambda^{2}-B_{1}}+\frac{A_{2}\lambda^{2}}{\lambda^{2}-B_{2}}+\frac{A_{3}\lambda^{2}}{\lambda^{2}-B_{3}}$$

where nsi occurs to be the refractive index of silica (fused) and λ is wavelength associated in μm scale.

The value of Sellmeier coefficients $A_{1}$, $A_{2}$, $A_{3}$, $B_{1}$, $B_{2}$ and $B_{3}$ in Eqn. (1) are taken from Ref. [39]. The fluctuation in the RIs of fused silica with temperature is just 1.28 × 10^−5^ (per degree Celsius). Thus, in normal contexts devoid of significant temperature variations, the influence of temperature is neglected. The plasmonic sensor's detecting performance is strongly sensitive on the plasmonic material. Gold (Au) was selected to be plasmonic material for its stability in terms of oxidation and having prominent resonant peaks.

The model of Drude-Lorentz determines the dielectric functional value of gold [39]:

(2) $$\varepsilon_{Au}=\varepsilon_{\infty }-\frac{{\omega_{D}}^{2}}{\omega \left(\omega +j\gamma_{D}\right)}-\frac{\Delta \varepsilon .\Omega_{L}^{2}}{\left(\omega^{2}-\Omega_{L}^{2}\right)+j\Gamma_{L}\omega }$$

where ε_Au_ is permittivity of gold and ε_∞_ = 5.9673 is permittivity at a high frequency. Numeric values of remaining parameters in Eqn. (2) are taken from Ref. [40]. As substitute for gold, AZO was later used as main plasmonic material. Relative permittivity of AZO (2% weight Al) is determined by the equation in the wavelength region 0.35 μm–2 μm, as given below [41]:

(3) $$\varepsilon AZO=\varepsilon b-\frac{\omega p^{2}}{\omega \left(\omega +j\gamma p\right)}+\frac{f1\omega 1^{2}}{\left(\omega 1^{2}-\omega^{2}-j\omega \gamma 1\right)}$$

where core electrons' polarization response (background permittivity) is indicated by *ε*_*b*_, the frequency (plasma) is denoted by *ω*_*p*_, and the Drude relaxation rate is denoted by *γ*. These parameters mentioned in Eqn. (3) have values: *ε*_*b*_ = 3.5402, *ω*_*p*_ = 1.7473 eV, *γ*_*p*_ = 0.04486 eV, *γ*_*1*_ = 0.1017, *f*_*1*_ = 0.5095 eV, and *ω*_*1*_ = 4.2942 eV.

Between the silica glass and the Au layer lies a thin TiO_2_ layer which provides the required adhesiveness. Because of its higher RI and transition metal behavior, TiO_2_ layer generates a tremendous number of extra electrons at its surface. Waves from the core are drawn into strong contact with SPP mode by a huge evanescent wave [40].

The refractive index of TiO_2_ can be calculated from the following equation [35]:

(4) $$nt=\sqrt{5.913+\frac{2.441\times 10^{7}}{\lambda^{2}-0.803\times 10^{7}}}$$

where *n*_*t*_ denotes TiO_2_'s refractive index and *λ* is the operational wavelength in the μm scale in Eqn. (4). It is critical for the plasmonic sensor that the Au and TiO_2_ layers are deposited sequentially on the exterior surface of PCF.

### 2.2 Simulation theory

COMSOL Multiphysics version 5.3a has been used as software for design and performance research. FEM is used to quantitatively investigate the proposed sensor's performance. To increase performance accuracy and element quality during simulation, a very small mesh element size with emfw domain is used. The mesh elements having properties: entire mesh area:164.2 μm^2^, average element quality: 0.8596, ensuring greater simulation accuracy. There are 125,806 triangular, 132 vertex, and 6564 edge elements, respectively. The interaction of a metal surface with an evanescent field produces a surface plasmon wave. During the advancement of surface plasmon wave, the frequency of SPP mode & fundamental core mode converge. The efficacy of the sensor is evaluated using confinement loss as the key criterion. When combined with a high sensitivity, a low confinement loss (CL) value can result in an ultrahigh sensor resolution. The performance of the sensor is judged using the CL characteristics of numerous analytes, which are determined using the equation below [42]:

(5) α=8.686×k0×Im(neff)×104(dB/cm)

where *k*_*0*_ *= 2π/λ* in Eqn. (5) denotes the free space wave propagation number, is the functioning wavelength, and Im (*n*_*eff*_) is the imaginary part of the effective mode index. For wavelength interrogation method, the sensitivity of the PCF sensor may be determined in nm/RIU using the expression:

(6) $$S_{\lambda }\left(nm/RIU\right)=\frac{\Delta \lambda peak}{\Delta na}$$

where, *Δλ*_*peak*_ indicates the change in resonance peaks and *Δη*_*a*_ indicate a difference in RI between two analytes.

AS is more efficient in terms of manufacturing cost than WS [39] when comparing the two sensing techniques. As a result, we tended to concentrate on AS enhancement in our work. When the signal is tightly coupled to a particular location on the surface, the AS rises concurrently. Equation [43] can compute the AS of the proposed sensor as follows:

(7) $$AS=-\frac{1}{\alpha \left(\lambda ,na\right)}\frac{\partial \alpha \left(\lambda ,na\right)}{\partial na}\left({RIU}^{-1}\right)$$

where*, α(λ, η*_*a*_*)* indicates loss during propagation at specific analyte RI and *∂α(λ, η*_*a*_*)* is the measure for CL difference for two consecutive *η*_*a*_. Detection of fractional changes in a refractive index is determined by sensor resolution. Sensor resolution is a significant performance aspect in investigating the detection accuracy of analyte RIs with the least possible RI variability. The equation used to determine resolution is [44]:

(8) $$SR=\frac{\partial \lambda \;\min\times \partial na}{\partial \lambda peak}\left(RIU\right)$$

Assuming *Δλ*_min_ = 0.1 nm, maximum calculated AR (amplitude resolution) in Eqn. (8) is 2.74 × 10^−6^ RIU & 2.47 × 10^−6^ RIU for x-pol and y-pol respectively for analyte RI 1.42. Consequently, the suggested sensor has the capability of detecting 10^−6^ orders of fluctuation in analyte RIs. It should be mentioned that noise from outside sources has been removed from the resolution measurement. Thus, the resolution of this research is the maximum possible resolution predicted with no instrumental noise from external disruptions. This resolution is referred to as ‘theoretical maximum resolution’ [45]. Also, the highest WR (wavelength resolution) came to be 36,500 and 40,500 for x-pol and y-pol respectively at analyte RI 1.42. Sensor performance is dependent on the range and strength of its signal to noise ratio (SNR). With a high SNR, the standard deviation is lower. In relation to this concept, the term ‘Figure of Merit’ (FOM) is used. FOM is also a critical performance parameter for calculating a sensor's detection limit, and may be expressed using the following equation [46]:

(9) $$FOM=\frac{S_{\lambda }\left(nm/RIU\right)}{FWHM\left(nm\right)}$$

Typically, this is expressed as sensitivity/full-width-half-maximum (sensitivity/FWHM). A higher FOM value suggests a more sensitive detection limit. As implied from the equation, as sensitivity increases with lowering FWHM, a large FOM may be achieved.

## 3 Sensing performance

To assess the suggested sensor's performance, both x-pol & y-pol propagation mode is evaluated. Performance analysis is critical for accurate visualization and execution of the proposed PCF sensor. The study considers performance factors such as amplitude sensitivity, wavelength sensitivity, sensor resolution, linearity, and figure of merit. The suggested plasmonic sensor is numerically analyzed by optimizing several geometrical parameters, including the thickness of Au (*t*_*g*_), TiO_2_ (*t*_*t*_) films, the pitch (*p*) size & the size of air holes *d*_*1*_.

The parameters were tuned first and we achieved an enhanced sensing response with *t*_*g*_ = 20 nm, *p* = 1.0 μm, *t*_*t*_ = 10 nm, & *d*_*1*_ = 0.90 μm. It is worth mentioning that in order to assess the impact of performance variation, just one parameter is changed while the others remain constant.

### 3.1 Distributions of electromagnetic fields and phase matching

The guided evanescent field principle is used by the PCF-based SPR sensor [1]. The efficiency with which the evanescent field travels from the center to the cladding of a metal can be maximized through careful design. Propagating light excites surface electrons in the metallic layer as it comes into touch with it. When the frequency of the center directed fields is the same as the frequency of the surface electrons, surface plasmon waves (SPWs) are created. The refractive index of their environment greatly affects the performance of these SPWs. Thus, local SRIs may be readily recognized by monitoring amplitude, resonance wavelength, as well as spectral mode shifts [44,47,48].

**Fig. 2:**
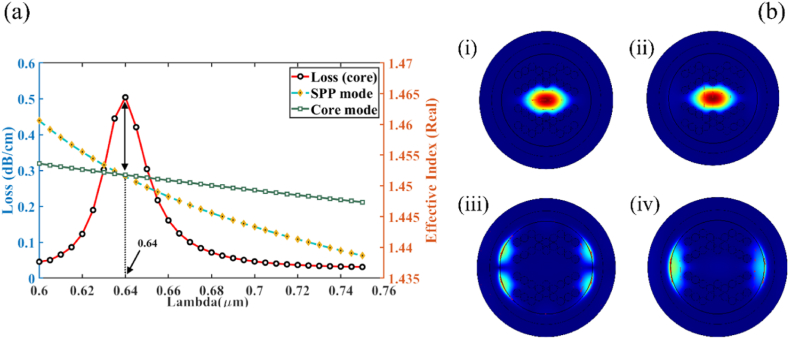
(a) Dispersion curve for core mode & SPP mode for analyte RI of 1.37; (b) Spatial configuration of the electromagnetic field at n_a_ = 1.37 for **(i, ii)** core mode for x-pol & y-pol respectively and **(ii, iv)** SPP mode x-pol & y-pol respectively.

The core mode's effective RI steadily decreases when the wavelength of a specific analyte is increased. The dispersion relation in y-polarization mode for analyte RI 1.37 is depicted in Fig. 2 (a), which presents curves of core mode (green line) and SPP mode (yellow line). Observations were made regarding the effective refractive indices (real) for two modes-core & SPP, in relation to a variety of frequencies. After plotting the actual values of the refractive indices (real) against operating wavelengths, a curve representing the transition between core and SPP mode was obtained. While both polarized modes exhibit high coupling, the y-pol is significantly stronger than the x-pol. 640 nm for y-pol is the wavelength where the effective index of core-guided and SPP modes meet, resulting in the peak loss (red line), which is referred to as phase matching. Here, a significant portion of energy is transferred from core-guided mode to SPP mode. The proposed sensor guiding properties and the coupling between the core guided and SPP modes are illustrated in Fig. 2 (b). The figures (i) and (ii) illustrate the distribution of the electric field in the core guided mode at analyte RI 1.37 for the x & y-polarized mode, while (iii) and (iv) illustrate the distribution of the electric field in SPP mode for the x & y-polarized mode, respectively at the same RI 1.37.

### 3.2 Optimization of Au and TiO_2_ thickness

**Fig. 3:**
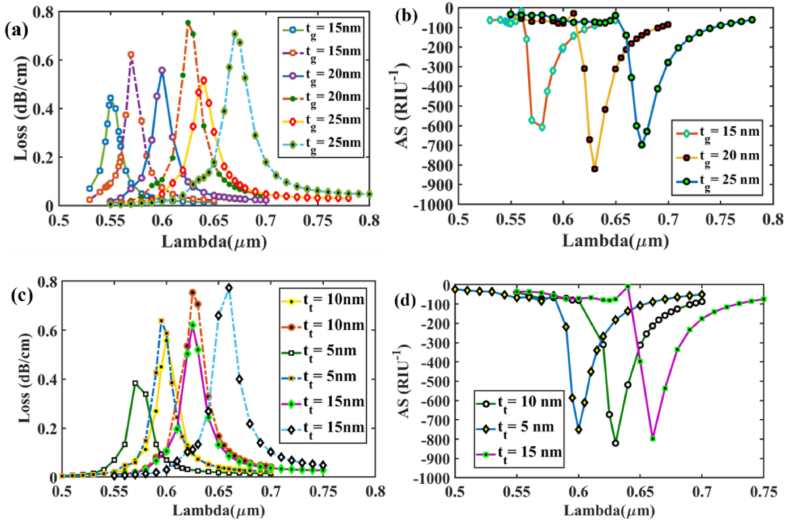
[X-polarization] measurements shown at 15, 20, and 25 nm for gold layer thickness, **(a)** variation in confinement loss for analyte RI of 1.37 **(solid lines)** & 1.38 **(dashed lines)**; **(b)** Sensitivity for amplitude variance shown for RI 1.37; measurements shown at 10, 5, and 15 nm for TiO_2_ layer thickness **(c)** variation in confinement loss for analyte RI of 1.37 **(solid lines)** & 1.38 **(dashed lines)**; **(d)** Sensitivity for amplitude variance shown for RI 1.37

Sensor performance is greatly affected by the fluctuation of the gold layer, as the metal layer generates surface plasmon waves. Due to damping effects, the depth of loss decreases as the gold layer thickness increases [48]. Loss depth & amplitude sensitivity for the difference in gold thickness at analytes RI 1.37 and 1.38 for x polarized mode are depicted in Fig. 3 (a) and (b). At analyte RI 1.37 (solid lines) & 1.38 (dashed lines), the greatest loss depth is 0.557 dB/cm and 0.753 dB/cm respectively, for x polarized mode & a 20 nm thickness. However, at *t*_*g*_ = 25 nm, the lowest loss depths of 0.442 dB/cm and 0.622 dB/cm for x-polarized mode are detected. Analyzing plots in Fig. 3 (a), we observe the CL spectrum exhibits a red-shift response as *t*_*g*_ is increased from 15 to 25 nm. A thin Au layer results in a high coupling interaction of the core and SPP modes, whereas a larger Au layer results in a weak coupling and a considerable reduction of CL depth. Also, in Fig. 3 (b), the AS peaks are located at about 608, 820, and 699 RIU^−1^ for gold thicknesses of 15, 20, and 25 nm in respective order. The maximum AS is attained at *t*_*g*_ = 20 nm at *n*_*a*_ = 1.37. Fig. 3 (c) and (d) illustrate loss depth & amplitude sensitivity for x-polarized mode, respectively, to changes in TiO_2_ thickness. A similar pattern has been seen for *t*_*t*_ and *t*_*g*_ variation. With 5, 10, and 15 nm of *t*_*t*_, the highest CL peaks are about 0.383, 0.557 and 0.619 dBcm^−1^ for RI 1.37 and 0.637, 0.753, and 0.772 dBcm^−1^ for RI 1.38. The linked AS peaks are measured approximately 820, 751 and 798 RIU^−1^ at RI analyte = 1.37.

**Fig. 4:**
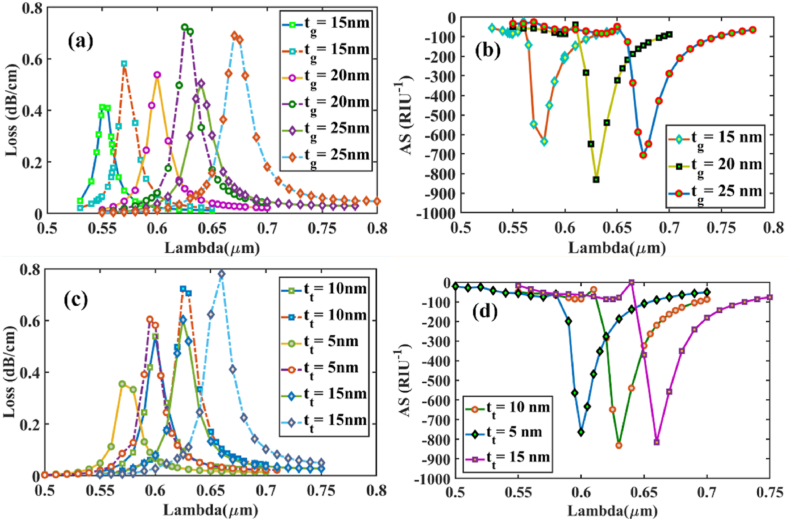
[Y-polarization] measurements shown at 15, 20, and 25 nm for gold layer thickness, **(a)** variation in confinement loss for analyte RI of 1.37 **(solid lines)** & 1.38 **(dashed lines)**; **(b)** Sensitivity for amplitude variance shown for RI 1.37; measurements shown at 10, 5, and 15 nm for TiO_2_ layer thickness **(c)** variation in confinement loss for analyte RI of 1.37 **(solid lines)** & 1.38 **(dashed lines)**; **(d)** Sensitivity for amplitude variance shown for RI 1.37

Similarly, in Fig. 4 (a), (b), (c) and (d) for y-polarized mode, loss depth and amplitude sensitivity for the difference in Au and TiO_2_ thickness between analytes RI 1.37 and 1.38 is shown. The AS peaks are located at about 635, 831, and 706 RIU^−1^ for *t*_*g*_ = 15, 20, and 25 nm, respectively. Meanwhile, for TiO_2_, the AS peaks- 762, 831, and 815 RIU^−1^ for *t*_*t*_ = 5, 10, and 15 nm, respectively confirm *t*_*g*_ = 20 nm and *t*_*t*_ = 10 nm optimized for both x-polarized and y-polarized mode for this design.

### 3.3 Optimization of air hole diameter (d_1_) and pitch (p) size

**Fig. 5:**
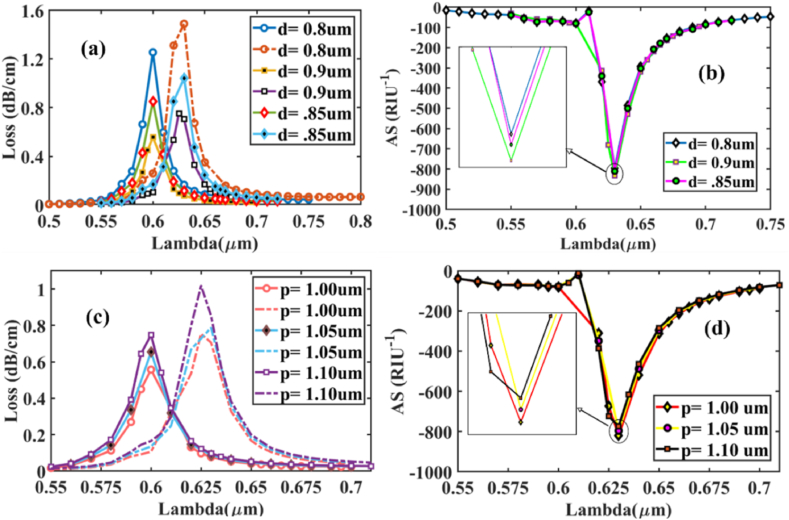
Varying diameter- 0.8 μm, 0.9 μm, and 0.85 μm, **[X-polarization] (a)** RI of 1.37 with CL spectrum variation **(solid lines)** and for RI of 1.38 **(dashed lines)**; **(b)** Sensitivity value for amplitude at RI of 1.37; Distinct pitch thickness- 1.0, 1.05, and 1.10 μm **(c)** RI of 1.37 with CL spectrum variation **(solid lines)** and for RI of 1.38 **(dashed lines)**; **(d)** Sensitivity value for amplitude at RI of 1.38.

Fig. 5 (a) illustrates the effect of uniform circular air hole diameter *d*_*1*_ change on the CL spectra at *n*_*a*_ 1.37 & 1.38 for x-polarized mode. It is obvious to see that as *d*_*1*_ increases from 0.8 μm to 0.9 μm, the CL curves decrease with values of 1.255, 0.85, and 0.557 dB/cm for *d*_*1*_ = 0.8, 0.85, and 0.9 μm respectively. Resonant Wavelength (RW), on the other hand, showed no variance. Additionally, the AS peaks are achieved at approximately 820 (0.9 μm), 810 (0.85 μm), and 795 (0.8 μm) RIU^−1^ for analyte RI 1.37, as indicated in Fig. 5 (b). Though the CL peaks at *d*_*1*_ = 0.8 μm, the AS is maximum at *d*_*1*_ = 0.9 μm. As a result, we identified 0.9 μm as the optimized air hole diameter for this design. Additionally, it is crucial to analyze the pitch (*p*) variation effect, which has a noticeable effect on sensing performance. To achieve precise sensing performance, we thoroughly calibrated the size, progressively increasing from 1 μm to 1.10 μm. CL spectra variation for *n*_*a*_ at 1.37 & 1.38 x-polarized mode shown in Fig. 5 (c). Pitch modification has almost no influence on the shifting of RW. Obtained AS peak values from Fig. 5 (d) are 820 (1.0 μm), 796 (1.05 μm), and 773 (1.10 μm) RIU^−1^ respectively. AS maximum value has been obtained for *p* = 1.0 μm considered as the optimized pitch layer thickness.

**Fig. 6:**
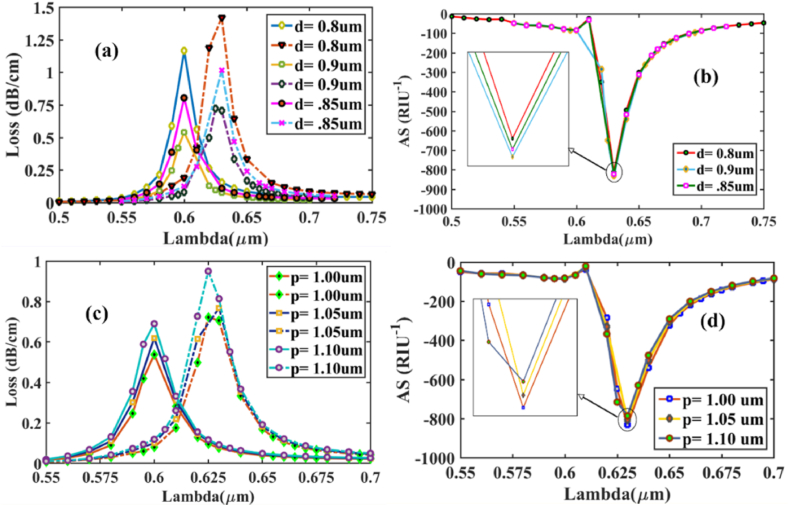
Varying diameter- 0.8 μm, 0.9 μm, and 0.85 μm, **[Y-polarization] (a)** RI of 1.37 with CL spectrum variation **(solid lines)** and for RI of 1.38 **(dashed lines)**; **(b)** Sensitivity value for amplitude at RI of 1.37; Distinct pitch thickness- 1.0, 1.05, and 1.10 μm **(c)** RI of 1.37 with CL spectrum variation **(solid lines)** and for RI of 1.38 **(dashed lines)**; **(d)** Sensitivity value for amplitude at RI of 1.38.

Similarly, in Fig. 6 (a), (b), (c) and (d) for y-polarized mode, loss depth and AS for the variation of *d*_*1*_ and *p* between analytes RI 1.37 and 1.38 is shown. The AS peaks are located at about 831, 822 and 811 RIU^−1^ for *d*_*1*_ = 0.9, 0.85, and 0.8 μm respectively. Meanwhile, for pitch, the AS peaks- 831, 809, and 785 RIU^−1^ for *p* = 1.0, 1.05, and 1.10 μm respectively, confirming *d*_*1*_ = 0.9 μm and *p* = 1.0 μm for this design.

### 3.4 The effect of analyte RI variation on sensing performance

**Fig. 7:**
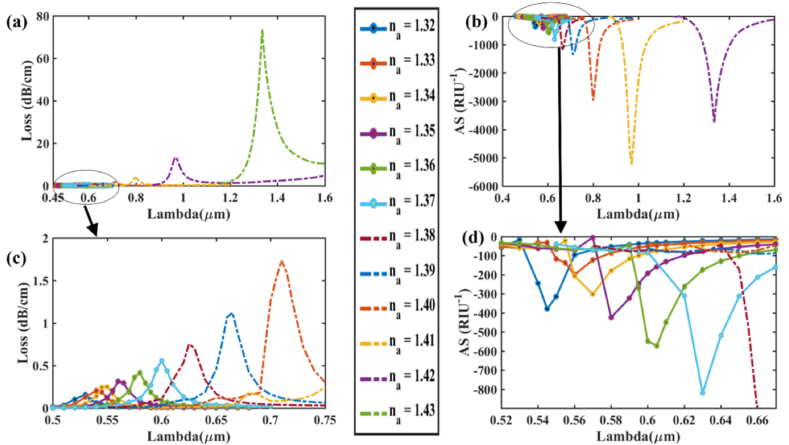
For Au **[x-polarization] (a)** Fundamental loss curves for RI 1.32–1.43; **(b)** curves of AS in RI range 1.32–1.42 for optimized parameters; **(c)** Zoomed portion showing fundamental core loss variation and **(d)** Zoomed portion showing sensitivity of amplitude in marked portion.

**Fig. 8:**
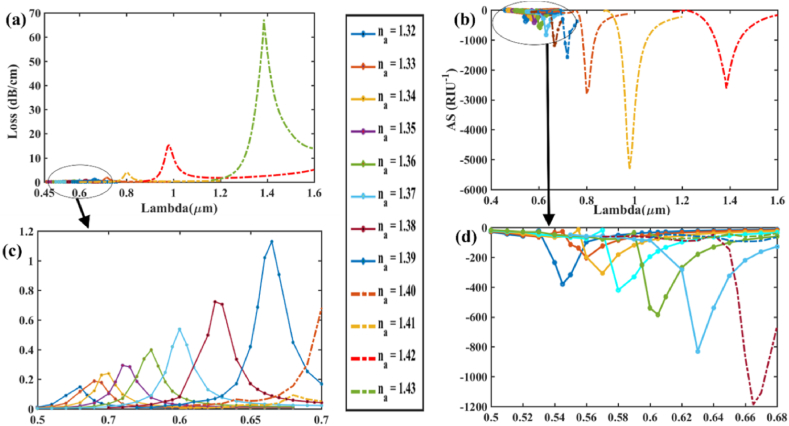
For Au **[y-polarization] (a)** Fundamental loss curves for RI 1.32–1.43; **(b)** AS curves in RI range 1.32–1.42 for optimized parameters; **(c)** Zoomed portion showing fundamental core loss variation and **(d)** Zoomed portion showing sensitivity of amplitude in marked portion.

Now that the optimal geometrical parameters for our proposed sensor have been determined, investigation on the effect of modifying the refractive index on the CL and AS is done. Fig. 7 (a) and (b) illustrate the effect of varying the RI of the analyte on CL and AS respectively, in the x-polarization mode. Any little fluctuation in the analyte RI produces a change in the *n*_*eff*_ (real) of the SPP mode, resulting in a blue or red-shift of the resonant wavelength [49]. Loss and sensitivity calculation is performed for varied *n*_*a*_ using Eqns. (5), (7). As shown in Fig. 7 (a), when RI fluctuates between 1.32 and 1.43, the RW peak moves from a shorter to a longer wavelength. Small changes in *n*_*a*_ may result in a large decrease in the RIs difference between the plasmonic and core modes. Thus, the cladding region receives most of the evanescent light rather than the core region. Consequently, light coupling across the metallic surface is improved, allowing it to interact with the dielectric channel more effectively. As a result, the CL spectrum gradually increases and causes red-shift. Fig. 7 (a) and (b) show the effect of changing the analyte's RI on CL and AS in the x-polarization mode while Fig. 7 (c) and (d) show zoomed portion for the loss variation, amplitude sensitivity respectively. For *n*_*a*_ = 1.32 & 1.33, respectively, the CL spectra were around 0.159 and 0.188 dBcm^−1^ at wavelengths of 0.53 and 0.54 μm. So, based on Eqn. (6), wavelength sensitivity is determined 1000 nm/RIU. Additionally, the maximum spectra of loss found to be around 13.32 and 73.85 dBcm^−1^ at RWs 0.97 and 1.335 μm, respectively, for *n*_*a*_ = 1.42 & 1.43. For maximum 365 nm RW shift, maximum WS is found to be 36,500 nm/RIU for this sensor in x-polarization as per Eqn. (6). Varying analyte RI from 1.32 to 1.43, the resonance wavelengths found are 530, 540, 550, 560, 580, 600, 625, 665, 710, 800, 970, and 1135 nm respectively. Again, in Fig. 8 (a) and (b), the effect of changing the analyte's RI on CL and AS in the y-polarization mode is demonstrated while Fig. 8 (c) and (d) show zoomed portion for the loss variation, amplitude sensitivity respectively. For RI variation range 1.32–1.43, resonance wavelengths found are 530, 540, 550, 560, 580, 600, 625, 665, 715, 800, 980 and 1385 nm respectively. In this case, for *n*_*a*_ = 1.32 & 1.33, respectively, CL spectra were around 0.149 and 0.177 dBcm^−1^ at wavelengths of 0.53 and 0.54 μm. So, based on Eqn. (6), wavelength sensitivity is determined 1000 nm/RIU. The maximum CL spectra found to be around 15.36 and 67.26 dBcm^−1^ at RWs of 0.98 and 1.385 μm, respectively, for *n*_*a*_ = 1.42 & 1.43. Highest WS is found to be 40,500 nm/RIU for maximum 405 nm RW shift.

For AS at RI = 1.41, loss peaks for RW 970 nm change from loss value of 0.248 dB/cm to 13.321 dB/cm at RI = 1.42. As a result of this difference in loss peak shifts for RI variation, the amplitude sensitivity is calculated as per Eqn. (7) and found 5268.76 RIU^−1^ for x-pol. Similarly, for y-pol AS is found 5335.32 RIU^−1^ as loss peaks for RW 980 nm change from loss value of 0.282 dB/cm at RI = 1.41–15.36 dB/cm at RI = 1.42. For RI = 1.42, the amplitude sensitivity was found 3775 RIU^−1^ for x-pol while it was 2624 RIU^−1^ for y-pol. For x-pol, at RW 1335 nm shows change in loss value from 1.90 dB/cm to 73.84 dB/cm and similarly for y-pol, RW 1385 nm shows change in loss value from 2.469 dB/cm to 67.26 dB/cm when shifting from RI = 1.42 to RI = 1.43. Thus, at *n*_*a*_ = 1.41, the sensor's maximal amplitude sensitivity is estimated around 5336 RIU^−1^.

Real-time biological sample testing is possible with analyte RI values between 1.32 and 1.43. According to previously published publications [42,44] these sensors operate within the same RI range. The RI range is substantially wider in this design, which results in a high degree of detection accuracy when sensing unknown chemical analytes. FOM is an important parameter for optimizing a sensor's limit of detection. The high FOM value reflects the sensor's superior performance. Thus, using Eqn. (9), the maximum FOM is approximately 744 & 773 RIU^−1^ for x-polarized & y-polarized mode. Table 1 shows detailed numerical results for Au & Table 2 shows the overall performance comparison of our proposed sensor to other previous works.

**Table 1:** Detailed performance analysis of the sensor for different RI using Au as plasmonic material.

RI	AS (RIU^−1^)		Amp. Res. (RIU) (× 10^−6^)		WS(nm/RIU)		Wav. Res. (RIU) (× 10^−6^)		FWHM		FOM	
	x pol	y pol	x pol	y pol	x pol	y pol	x pol	y pol	x pol	y pol	x pol	y pol
1.32	215.4	215.9	46.4	46.3	1000	1000	100	100	19.57	18.74	51	53
1.33	255.8	259.8	39.1	38.5	1000	1000	100	100	20.31	19.38	49	52
1.34	342.0	343.7	29.2	29.1	1000	1000	100	100	18.90	18.24	52	55
1.35	423.4	420.8	23.6	23.8	2000	2000	50	50	19.35	19.57	103	102
1.36	548.0	585.1	18.2	17.1	2000	2000	50	50	18.61	25.82	107	77
1.37	820.7	831.2	12.2	12.0	2500	2500	40	40	19.84	19.73	126	127
1.38	1178	1187	8.49	8.43	4000	4000	25	25	21.71	22.06	184	181
1.39	1359	1570	7.36	6.37	4500	5000	22.2	20	23.87	23.66	188	211
1.40	2982	2882	3.35	3.47	9000	8500	11.1	11.8	30.05	27.25	299	311
1.41	5269	5336	1.9	1.87	17,000	18,000	5.88	5.56	34.03	35.23	500	510
1.42	3775	2624	2.65	3.81	36,500	40,500	2.74	2.47	49.03	52.40	744	773
1.43	–	–	–	–	–	–	–	–	–	–	–	–

**Table 2:** Comparative study of the performance with existing PCFs as RI sensor for plasmonic material Au.

Refs		Range	AS (RIU^−1^)	Amplitude Resolution (RIU)	WS (nm/RIU)	Wavelength Resolution (RIU)	Figure of Merit (FOM)
[42]		1.32–1.41	1170		34,000		310
[50]		1.413–1.415	1266.67		50,000	4.00 × 10^−4^	–
[51]		1.385–1.40			10,000	2.00 × 10^−5^	–
[52]		1.33–1.42	4358.09	2.29 × 10^−6^	21,000	4.76 × 10^−6^	729
[53]		1.35–1.41	4738.9	2.11 × 10^−6^	14,500	6.90 × 10^−6^	387
[25]		1.40–1.44	1739.26	5.75 × 10^−6^	9600	1.04 × 10^−5^	–
[1]	x pol	1.33–1.38	371.5	2.69 × 10^−5^	4300	2.32 × 10^−5^	–
	y pol		420.4	2.37 × 10^−5^	4600	2.17 × 10^−5^	–
This paper	x pol	1.32–1.42	5269	2.65 × 10^−6^	36,500	2.74 × 10^−6^	744
	y pol		5336	3.81 × 10^−6^	40,500	2.47 × 10^−6^	773

Because of its potential uses in real-time detection of biochemical and biological materials, the RIs range spanning 1.32 to 1.43 was the focus of this numerical study. Within this measurement range, the optical structural parameters and the thickness of the metal layer are tuned for best results. Mucosa (human intestine) = 1.329–1.338, acetone = 1.36, urine (human body) = 1.341–1.346, 10% glucose + water = 1.347, 20% glucose + water = 1.3635, ethanol = 1.361, human liver = 1.369, and numerous cancerous cells like HeLa, Jurkat possess RI values that fall within the prescribed range. It is worth noting that the suggested sensor's detecting range is nearly equal to that of previously described plasmonic RI sensors [42,44,54].

### 3.5 The effect of improved RW shift and increased RI range by using AZO as plasmonic material

Transparent conducting oxides (TCO) have recently been hailed as a promising alternative to the existing plasmonic metals due to their metal-like conductivity and ability to be utilized as ultrathin films in the near-infrared and visible regions [[55], [56], [57]]. The researchers [51] examined the performances of AZO (2% wt. of Al in ZnO), ITO & Gallium doped with ZnO which forms GZO, and concluded that Aluminum-doped Zinc Oxide (AZO) had the lowest loss and the most adjusting possibilities for specific applications in terms of carrier concentrations and doping. Thus, it is justified that AZO is a realistic choice for plasmonic material and is capable of being employed effectively in sensing applications. A characteristic of AZO for applications that require sensing is that it does not need coating metals on the inner surface of PCF holes or filling gaps, also just a small quantity of analyte is required for RI monitoring. Sharp, narrow, and distinct loss peak red-shifts are observed for the CL spectrum when AZO substitutes Au as plasmonic material. Due to the near-IR plasma frequency provided by AZO's low charge carrier density, a wide spectral range of RI detection may be feasible. For this work, analyte RI is differed from 1.19 to 1.42 which gives superior ultra-wide range of detection for various liquid analytes like Sevoflurane [58] as well as gaseous compounds including organics containing fluorine & liquid CO_2_ [59].

**Fig. 9:**
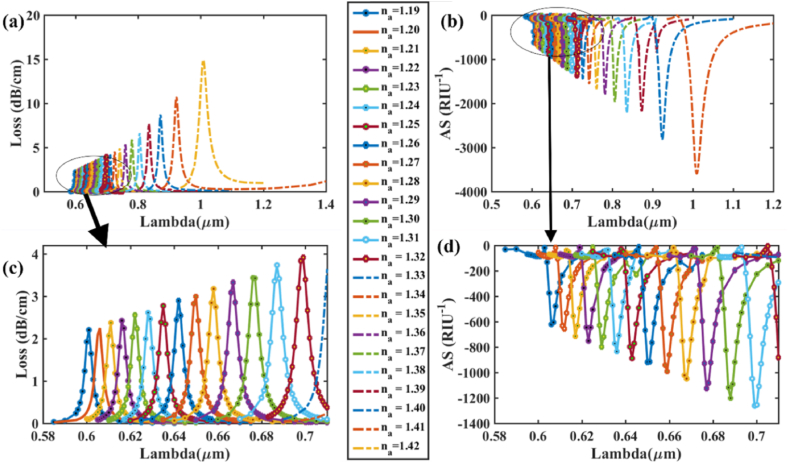
For AZO **[x-polarization] (a)** Fundamental loss curves for RI of 1.19–1.42; **(b)** AS curves in RI range 1.19–1.41 for optimized parameters; **(c)** Zoomed portion showing fundamental core loss variation and **(d)** Zoomed portion showing sensitivity of amplitude in marked portion.

**Fig. 10:**
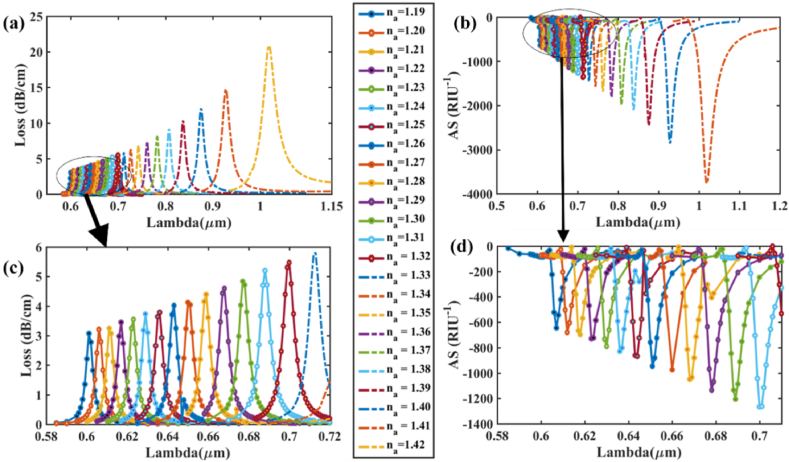
For AZO **[y-polarization] (a)** Fundamental loss curves for RI of 1.19–1.42; **(b)** AS curves in RI range 1.19–1.41 for optimized parameters; **(c)** Zoomed portion showing fundamental core loss variation and **(d)** Zoomed portion showing sensitivity of amplitude in marked portion.

**Fig. 11:**
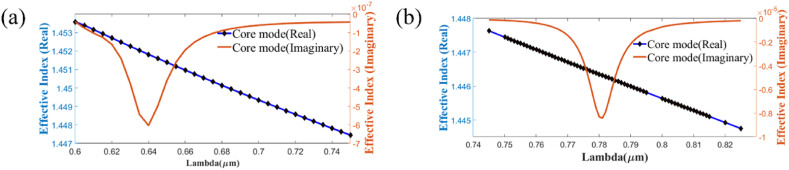
[x-polarization] (a) Real & imaginary refractive index values for Gold **(b)** Real & imaginary refractive index values for AZO

Fig. 9 (a) & (b) show the effect of varying the analyte RI on CL and AS respectively, in the x-polarization mode while Fig. 9 (c) and (d) show zoomed portion for the loss variation, amplitude sensitivity respectively. Highest CL spectra 14.911 dBcm^−1^ occurs for RI 1.42 where RW is 1.008 μm. For AS in Fig. 9 (b), at RI = 1.41, loss peaks for RW 1008 nm change from loss value of 0.408 dB/cm to 14.911 dB/cm at RI = 1.42. As a result of this difference in loss peak shifts for RI variation, the amplitude sensitivity is calculated as per Eqn. (7) and found 3593.08 RIU^−1^ for x-pol. Thus, with *n*_*a*_ = 1.41, the maximum AS comes 3593 RIU^−1^ while for the sensitivity for wavelength is 8500 nm/RIU as RW shifts from 923 nm at RI = 1.41–1008 nm at RI = 1.42 as per Eqn. (6). Maximum RW shift is calculated as 85 nm. Furthermore, the most sensitive amplitude resolution & wavelength resolution obtained is 2.78 × 10^−6^ RIU and 1.18 × 10^−5^ RIU respectively. The best value of FOM is evaluated as 413. Similarly, for the y-polarization mode, Fig. 10 (a) & (b) demonstrate the impact of altering the analyte RI on CL and AS, respectively while Fig. 10 (c) and (d) show zoomed portion for the loss variation, amplitude sensitivity respectively. At *n*_*a*_ = 1.42 and a RW of 1.018 μm, the highest CL spectra of 20.88 dB/cm occurs. Fig. 11 (a) & (b) shows the real and imaginary refractive index values for Gold & AZO respectively (x-pol). For AS in Fig. 10 (b), at RI = 1.41, loss peaks for RW 1018 nm change from loss value of 0.544 dB/cm to 20.879 dB/cm at RI = 1.42. As a result of this difference in loss peak shifts for RI variation, the amplitude sensitivity is calculated as per Eqn. (7) and found 3763.42 RIU^−1^ for x-pol. Thus, at *n*_*a*_ = 1.41, the sensor's maximum AS is estimated to be around 3763 RIU^−1^, while the WS is approximately 9100 nm/RIU as RW shifts from 927 nm at RI = 1.41–1018 nm at RI = 1.42 as per Eqn. (6). Maximum RW shift is calculated as 91 nm. Additionally, the maximum amplitude and wavelength resolutions that are attained: 2.66 × 10^−6^ RIU and 1.10 × 10^−5^ RIU, respectively. The peak value of FOM is determined to be 441 for y-polarized mode. Table 3 contains the detailed numerical results for AZO & Table 4 shows a comparison with existing works with AZO as plasmonic material. AZO (aluminum-doped zinc oxide) is a plasmonic material that operates at higher wavelengths, and since it has a low charge carrier density and plasma frequency, it can be operated in the NIR range if the range is to be expanded to encompass a wider spectrum of wavelengths. The major reason behind the significant drop in amplitude sensitivity (AS) is due to the low charge carrier density. Since AS depends on the loss difference between two consecutive RI which is very less in case of AZO mainly because AZO has a low amount of charge carrier which results in low confinement loss. The resultant loss is very low for AZO as compared to other plasmonic materials like gold or silver when operated at the same wide spectral range. Furthermore, wavelength sensitivity (WS) is greatly reduced due to very small change of resonant wavelength peaks. Compared to the difference of resonant peak shifts of 170 nm for Gold at RI = 1.41, the resultant peak change stands at 85 nm for AZO for x-pol. Thus, for the RI of 1.41, the wavelength sensitivity (WS) of Gold is 17,000 nm/RIU for x-pol mode while it is only 8500 nm/RIU for AZO. Jitendra Narayan et al. [60] proposed an AZO-coated PCF SPR sensor that operates at longer wavelengths between 1600 and 2000 nm and has a maximal wavelength sensitivity of 5000 nm/RIU for y-polarization mode.

**Table 3:** Detailed performance analysis of the sensor for different RI using AZO as plasmonic material.

RI	AS (RIU^−1^)		Amp. Res. (RIU) (× 10^−6^)		WS(nm/RIU)		Wav. Res. (RIU) (× 10^−6^)		FWHM		FOM	
	x pol.	y pol.	x pol.	y pol.	x pol.	y pol.	x pol.	y pol.	x pol.	y pol.	x pol.	y pol
1.19	620.6	644.1	16.1	15.5	500	500	200	200	4.15	4.21	120	118
1.20	656.1	679.6	15.2	14.7	500	500	200	200	4.44	4.24	113	118
1.21	715.2	698.3	14.0	14.3	500	600	200	167	4.24	4.46	118	134
1.22	753.1	729.4	13.3	13.7	600	600	167	167	4.54	4.46	132	134
1.23	797.6	788.9	12.5	12.7	600	600	167	167	4.61	4.64	130	129
1.24	834.2	830.6	12.0	12.0	700	700	143	143	4.81	4.76	145	147
1.25	889.7	868.1	11.2	11.5	700	700	143	143	4.94	5.00	142	140
1.26	919.2	947.4	10.9	10.6	800	700	125	143	5.12	5.13	156	136
1.27	993	981.8	10.1	10.2	800	900	125	111	5.32	5.42	150	166
1.28	1020	1050	9.8	9.53	900	900	111	111	5.31	5.56	169	161
1.29	1125	1136	8.89	8.80	900	900	111	111	5.67	5.86	158	153
1.30	1203	1206	8.31	8.29	1100	1100	90.9	90.9	6.11	12.51	179	87
1.31	1263	1265	7.92	7.90	1200	1200	83.3	83.3	6.39	6.37	187	188
1.32	1363	1362	7.33	7.34	1200	1300	83.3	76.9	6.87	6.80	175	191
1.33	1444	1465	6.93	6.82	1500	1400	66.7	71.4	7.30	7.32	205	191
1.34	1550	1575	6.45	6.35	1600	1600	62.5	62.5	7.84	7.74	204	206
1.35	1676	1687	5.97	5.93	1800	1800	55.6	55.6	8.48	8.38	212	214
1.36	1805	1813	5.54	5.52	2100	2100	47.6	47.6	9.17	9.23	229	227
1.37	1966	1990	5.09	5.02	2400	2600	41.7	38.5	10.24	10.28	234	252
1.38	2217	2105	4.51	4.75	3000	2900	33.3	34.5	11.50	11.49	261	252
1.39	2175	2456	4.60	4.07	3700	3800	27.0	26.3	13.19	13.39	280	284
1.40	2819	2851	3.55	3.51	5100	5200	19.6	19.2	15.84	15.85	322	328
1.41	3593	3763	2.78	2.66	8500	9100	11.8	11.0	20.56	20.65	413	441
1.42	–	–	–	–	–	–	–	–	–	–	–	–

**Table 4:** Comparative study of the performance with existing PCFs using plasmonic material AZO.

Refs		Range	AS (RIU^−1^)	Amplitude Resolution (RIU)	WS (nm/RIU)	Wavelength Resolution (RIU)	Figure of Merit (FOM)
[52]		1.31–1.39	3908	–	1700	5.88 × 10^−5^	792
[60]			167	–	5000	2.00 × 10^−5^	–
[61]		1.27–1.42	4602.67	–	7200	–	1118
This paper	x pol	1.19–1.42	3593	2.78 × 10^−6^	8500	1.18 × 10^−5^	413
	y pol		3763	2.66 × 10^−6^	9100	1.10 × 10^−5^	441

**Fig. 12:**
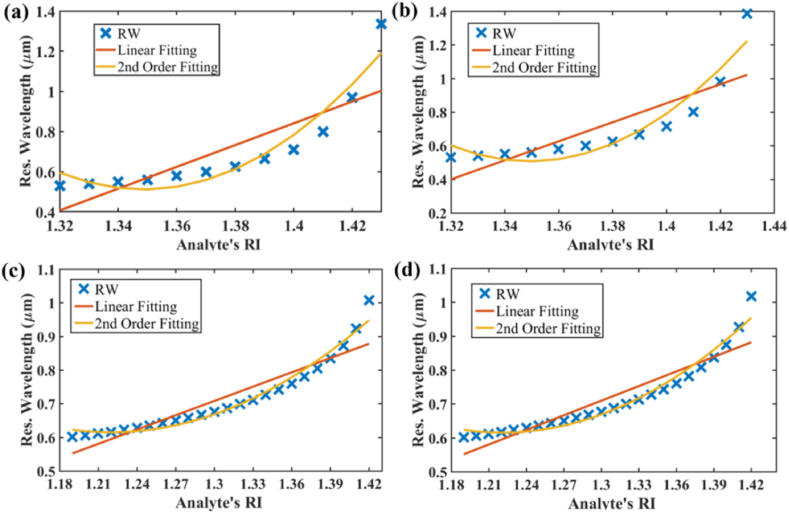
Fiber linearity of **[x-polarization] (a)** optimized parameters & Au; **[y-polarization] (b)** optimized parameters & Au; Linearity **[x-polarization]; (c)** optimized parameters & AZO; **[y-polarization] (d)** optimized parameters & AZO.

Fig. 12 (a) and (b) illustrate excellent 2nd order fitting curves of the resonance wavelength for Au which indicates the enhanced sensor's high accuracy for determining analyte refractive indices. Value of R^2^ is evaluated as 0.9140 and 0.9037 respectively. The fitting curves of 2nd order for AZO are shown in Fig. 12 (c) and (d) and equations with R^2^ value are mentioned in Table 5.

**Table 5:** Polynomial fitting equation obtained from simulation data along with R-square value for Gold and AZO.

Metrics	Gold	AZO
2nd Order Eqn. (x-pol)	$y=187.22-276.8932x+102.6598x^{2}$	$y=13.0343-20.3594x+8.3438x^{2}$
R^2^ value (x-pol)	0.9140	0.9720
2nd Order Eqn. (y-pol)	$y=200.66-296.6813x+109.9401x^{2}$	$y=13.351-20.8624x+8.5437x^{2}$
R^2^ value (y-pol)	0.9037	0.9692

### 3.6 Temperature sensing

The use of SPR-based PCF devices is on the rise in the field of temperature detection owing to the strong temperature dependence of the effective refractive index & confinement loss [62]. So, in addition to sensing analytes, our sensor can also be used to measure temperature if the analyte is sensitive to temperature. In proposed design, ethanol was used as analyte and Aluminum zinc oxide (AZO) as plasmonic material as it generated sharper peaks than gold (Au) as per numerical investigation. Ethanol is utilized as an analyte whose RI varies with temperature, whereas AZO is involved as a plasmonic material whose RI varies with the nature of light. It has been reported that Zinc Oxide is a suitable response candidate for toxic and hazardous gases like Ethanol and Acetone [[63], [64], [65]]. Zinc oxide is an n-type semiconductor with a large energy bandgap (Eg = 3.37eV), a large exciton binding energy (60 meV), high electron mobility, and exceptional chemical and thermal stability [66,67]. It has been reported that ZnO nanomaterials with various morphologies exhibited excellent sensitivities to reductive gases such as ethanol, CO, H_2_, and H_2_S. Gamal et al. mentioned AZO as plasmonic material to be provide better sensitivity for proposed multi-functional SPR biosensor [68].

Resonance wavelength shifts significantly as refractive index of ethanol varies with variation in temperature which is the basis of the temperature sensing. The equation used to figure out how RI changes with temperature is [69]:

(10) $$n_{1}=n_{0}+\frac{dn}{dT}\left(T_{1}-T_{0}\right)$$

Here, *n*_*0*_ and *n*_*1*_ represent refractive indices for temperatures *T*_*0*_ and *T*_*1*_, respective and *dn/dT* = −3.117 × 10^−4^ °C^−1^ is ethanol's thermo-optical coefficient. Refractive index of ethanol is *n*_*0*_ = 1.361 when temperature *T*_*0*_ is 20 °C.

Temperature sensitivity is usually determined by using equation [70]:

(11) $$S_{T}=\frac{\partial \lambda peak\left(T\right)}{\partial T}\left(nm/{}^{\circ}C\right)$$

where $S_{T}$, ∂T, ∂λpeak(T) mean temperature sensitivity, temperature variation & peak shift due to temperature variation respectively in Eqn. (11).

Ethanol manifests a boiling point and a melting point of 78.37 °C, −114.1 °C, respectively. In liquid state of ethanol, as the temperature changes, its RI alters from 1.343 to 1.403. Thus, the index of liquid ethanol range for any temperature aligned with our sensor's detection range. Therefore, our proposed sensor can detect minimum change in environmental temperature.

**Fig. 13:**
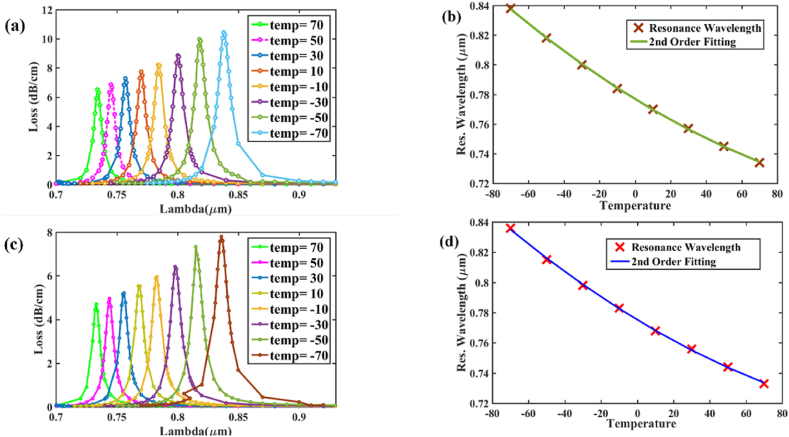
[y-polarization] (a) CL curves for temp ranging from 70 °C to −70 °C; **(b)** curve fitting for loss curve in (a) for temperature variation; **[x-polarization]; (c)** CL curves for temp ranging from 70 °C to −70 °C and **(d)** curve fitting for loss curve in (c) for temperature variation.

Fig. 13 (a), (c) shows as temperature decreases from 70 °C to −70 °C, RW shifts right and CL increases both for x-polarization and y-polarization which indicates that temperature can be easily detected with our sensor. The RW peak shifts when temperature is decreased which causes RI to increase as per calculation from Eqn. (10). This enables core mode power to leak greatly into the cladding area. Therefore, according to the RI limit of ethanol, it falls within the proposed sensor's range of detection. After light passes through the PCF sensor, the output light is analyzed by an analyzer which provides the resonance wavelength which is substituted in Eqn. (12) (x-pol) or Eqn. (13) (y-pol). Solving this equation, the temperature is determined.

Fig. 13 (b), (d) represent 2nd order fittings between different temperatures and its resonance wavelengths for x-pol (R^2^ = 0.9995) and y-pol (R^2^ = 0.9998) respectively.

The equation of the fitting curve for x-pol:

(12) $$\lambda resonance=0.77522-0.0007244\times T+1.8601\times 10^{-6}\times T^{2}$$

and for y-pol:

(13) $$\lambda resonance=0.77681-0.0007351\times T+1.875\times 10^{-6}\times T^{2}$$

here, *T* represents temperature and *λ*_*resonance*_ represents resonance wavelength in μm for that temperature. The spectrum analyzer can be used to get the value of the resonant wavelength substituting that value in either Eqn. (12) or Eqn. (13) depending on polarization, the temperature can be identified.

The maximum sensitivity we get is 1.05 nm/^°^C & 1 nm/^°^C and best resolution is 0.095 °C & 0.1 °C respectively for x-polarization and y-polarization. Sensitivity and other parameters at different temperatures are shown in Table 6, Table 7 respectively for x-pol and y-pol. It should be noted that our sensor's sensitivity can be increased a great amount only be replacing AZO by Au as plasmonic material. However, we have earlier chosen AZO to investigate our sensor performance because it provides sharp peaks at resonance wavelength which are easy to detect.

**Table 6:** Performance analysis of proposed sensor as Temperature sensor (x-pol).

Temp (^o^C)	Resonant wavelength (μm)	Sensitivity (nm/^o^C)	Resolution
−70	0.836	1.05	0.09524
−50	0.815	0.85	0.11765
−30	0.798	0.75	0.133333
−10	0.783	0.75	0.133333
10	0.768	0.6	0.166667
30	0.756	0.6	0.166667
50	0.744	0.55	0.181818
70	0.733

**Table 7:** Performance analysis of proposed sensor as Temperature sensor (y-pol).

Temp (^o^C)	Resonant wavelength (μm)	Sensitivity (nm/^o^C)	Resolution
−70	0.838	1	0.1
−50	0.818	0.9	0.111111111
−30	0.8	0.8	0.125
−10	0.784	0.7	0.142857143
10	0.77	0.65	0.153846154
30	0.757	0.6	0.166666667
50	0.745	0.55	0.181818182
70	0.734		

Different analyte RI and temperature sensing capabilities for Gold & AZO are shown in Table 8. In this paper, we propose a multipurpose sensor that can detect analytes as well as temperature. However, the sensor cannot simultaneously detect both. Further research scope can be attempted to detect both RI and temperature.

**Table 8:** Sensing capabilities for Gold & AZO.

Sensing Capability	Analyte under Investigation	Gold AS (RIU^−1^)	AZO AS (RIU^−1^)
RI		Range: 1.32–1.42	Range: 1.19–1.41
1.266	Sevoflurane	–	947.4
1.33	Methyl	259.8	1465
1.35	Milk	423.8	1687
1.36	Blood cells, Acetone	585.1	1813
1.37	Acetic Acid	831.2	1990
1.38	Skin Cell	1187	2217
1.39	Cancerous blood cells	1570	2456
1.401	Breast cancer cell	2882	2851
1.41	Decane [71]	5336	3763
1.42	50% Sugar solution	3775	–
**Temperature (**^**o**^**C)**	**Ethanol**	**Polarization**	**AZO Sensitivity (nm/**^**o**^**C)**
−70	Liquid Ethanol	x-pol	1.05
−50	Liquid Ethanol	y-pol	0.9

### 3.7 Double step dual peak shift sensitivity (DS-DPSS)

**Fig. 14:**
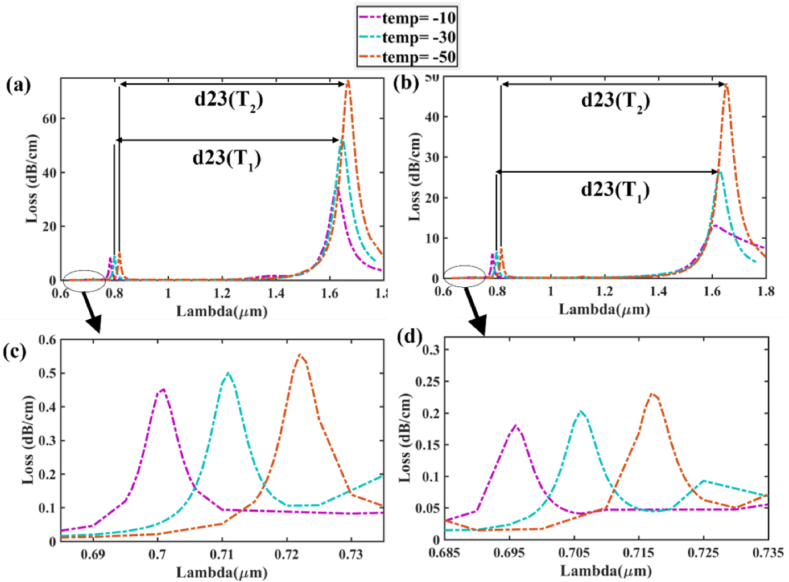
(a) Curves of 3 loss peaks due to temperature variation in y polarization.; **(b)** Curves of 3 loss peaks due to temperature variation in x-polarization; **(c)** Zoomed portion of 1st loss peak in y polarization; **(d)** Zoomed portion of 1st loss peak in x polarization.

It has been observed that our sensor exhibits three distinct loss peaks at three different resonance wavelengths for refractive indices that are obtained during varying temperature from −70 °C to 70 °C. From Fig. 14 it is visible that as temperature increases the distance between two consecutive peaks decreases. Utilizing this phenomenon, we can calculate dual peak shift sensitivity [DPSS] [61] for temperature in case of both 1st & 2nd peak and 2nd & 3rd peak as shown in Fig. 14 (a), (c) for y-pol & Fig. 14 (b), (d) for x-pol with zoomed portions which will able us to introduce a new method called “*DS-DPSS* “. In this method we will first find out the DPSS for 1st and 2nd peak and then for 2nd and 3rd peak, therefore by looking into both values we will determine the temperature. The equation used for DPSS is [61]:

(14) $$S_{DPSS}\left(T\right)=\frac{\left(\lambda p_{2},T_{2}-\lambda p_{1},T_{2}\right)-\left(\lambda p_{2},T_{1}-\lambda p_{1},T_{1}\right)}{T_{2}-T_{1}}\times 10^{3}\left(nm/{}^{\circ}C\right)$$

Where $S_{DPSS}\left(T\right)$ denotes double peak shift sensitivity of temperature, $\lambda p_{2},T_{2}$ & $\lambda p_{1},T_{2}$ denotes RW for 2nd peak & 1st peak respectively for temperature $T_{2}$. Similarly, $\lambda p_{2},T_{1}$ & $\lambda p_{1},T_{1}$ denotes RW for 2nd peak & 1st peak respectively for temperature $T_{1}$.

The values of distances between two consecutive peaks and DPSS are given in Table 7.

In Table 9, d_12_ is the distance between 1st peak and 2nd peak for a particular temperature and d_23_ is the distance between 2nd peak and 3rd peak for that temperature. S_12_ is the DPSS for 1st peak and 2nd peak and S_23_ is the DPSS for 2nd peak and 3rd peak for that temperature. The maximum DPSS is 0.35 nm/^°^C for y polarization and 0.3 nm/^°^C for x polarization.

**Table 9:** Analysis of DS-DPSS in Temperature sensing.

Temp (^o^C)	Pola-rization	d_12_	d_23_	S_12_	S_23_	Pola-rization	d_12_	d_23_	S_12_	S_23_
−50	x-pol	0.098	0.838	0.3	0.3	y-pol	0.096	0.849	0.35	0.2
−30		0.092	0.832	0.2	0.1		0.089	0.845	0.3	0.15
−10		0.088	0.83	–	–		0.083	0.842	–	–

The sensing technique in x polarization for determining −50 °C and −30 °C will be to follow the subsequent logic.

The distance between two peaks (sequentially 1st & 2nd peaks, then 2nd & 3rd peaks) of the same polarization is used to calculate the dual peak sensitivity. We use DS-DPSS to detect −50 °C temperature for y-pol.

$\lambda p_{2},T_{2}-\lambda p_{1},T_{2}$ denotes the distance between the 1st and 2nd peak for a particular temperature say *T*_*2*_ = −50 °C which is simply written as d_12_ (−50 °C) whereas $\lambda p_{2},T_{1}-\lambda p_{1},T_{1}$ denotes the distance between the 1st and 2nd peak for a particular temperature say *T*_*1*_ = −30 °C which is simply written as d_12_ (−30 °C).

As per Eqn. (14), we calculate *S*_*DPSS*_:

$$S_{DPSS}\left(T\right)=\frac{\left(\lambda p_{2},T_{2}-\lambda p_{1},T_{2}\right)-\left(\lambda p_{2},T_{1}-\lambda p_{1},T_{1}\right)}{T_{2}-T_{1}}\times 10^{3}\left(nm/{}^{\circ}C\right)\begin{array}{l} S_{12}=\frac{d_{12}\left(-50^{\circ }C\right)-d_{12}\left(-30^{\circ }C\right)}{-50^{\circ }C-\left(-30^{\circ }C\right)}\times 10^{3}\left(nm/{}^{\circ}C\right)=\frac{0.096-0.089}{-20}\times 10^{3}\left(nm/{}^{\circ}C\right)=-0.35nm/{}^{\circ}C\\ \left|S_{12}\right|=0.35nm/{}^{\circ}C \end{array}$$

$\lambda p_{3},T_{2}-\lambda p_{2},T_{2}$ denotes the distance between the 2nd and 3rd peak for a particular temperature say *T*_*2*_ = −50 °C which is simply written as d_23_ (−50 °C) whereas $\lambda p_{3},T_{2}-\lambda p_{2},T_{2}$ denotes the distance between the 2nd and 3rd peak for a particular temperature say *T*_*1*_ = −30 °C which is simply written as d_23_ (−30 °C).

$$\begin{array}{l} S_{23}=\frac{d_{23}\left(-50^{\circ }C\right)-d_{12}\left(-30^{\circ }C\right)}{-50^{\circ }C-\left(-30^{\circ }C\right)}\times 10^{3}\left(nm/{}^{\circ}C\right)=\frac{0.849-0.845}{-20}\times 10^{3}\left(nm/{}^{\circ}C\right)=-0.2nm/{}^{\circ}C\\ \left|S_{23}\right|=0.2nm/{}^{\circ}C \end{array}$$

Thus, if $\left|S_{12}\right|=0.35nm/{}^{\circ}C$ and $\left|S_{23}\right|=0.2nm/{}^{\circ}C$, then Temp = −50 °C.

So, within precision of ±0.10 to ±0.15 nm/^o^C, proposed sensor can realize this Double Step Dual Peak Shift Sensitivity to accurately determine the temperature for both x-pol and y-pol.

Along with the conventional method of identifying temperature where RW shift due to temperature variation is calculated, a new method is proposed to detect temperature variation using DS-DPSS in Fig. 14. Previously, M.R Islam et al. [61] introduced dual peak shift sensitivity (DPSS) for RI detection, whereas DS-DPSS technique is used by the proposed sensor for temperature detection. It is to be mentioned that DS-DPSS technique can also be applied in RI detection. Table 10 shows the comparison with existing Temperature sensors.

**Table 10:** Study of comparison with existing Temperature sensors.

Reference	Sensitivity (nm/^o^C)	Resolution (RIU)	Sensing range (^o^C)	DS-DPSS (nm/^o^C)
[8]	1.551	0.064	35 to 100	–
[69]	0.75	0.133	−70 to 70	–
[72]	0.229	0.437	25 to 55	–
[73]	0.978	0.102	25 to 100	–
[74]	1.25	0.08	−70 to 70	–
[75]	4.67	0.03	30 to 90	–
[76]	0.07	–	30 to 80	–
Our paper	1.05 (x-pol)	0.09524	−70 to 70	0.35
	1.00 (y-pol)	0.1		0.30

### 3.8 Tolerance investigation

**Fig. 15:**
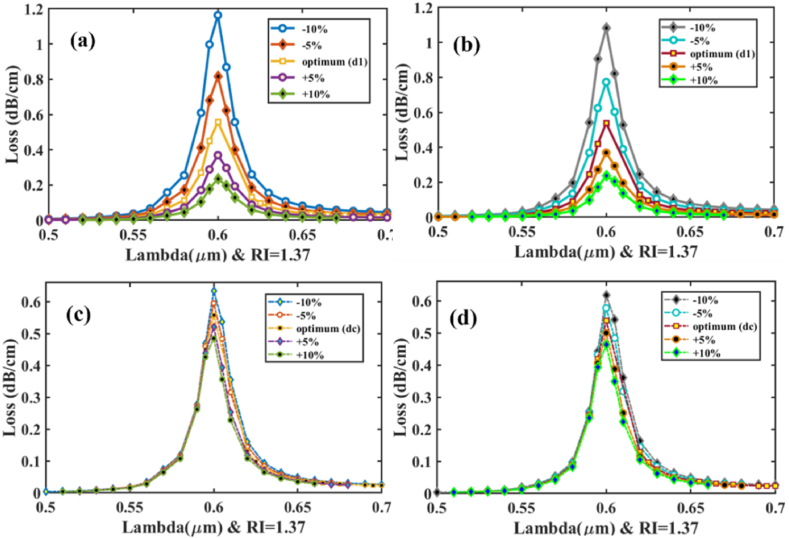
Fabrication tolerance analysis- Effects of CL **[x-polarization] (a)** diameter −10%, −5%, +10% and +5% of ***d***_***1***_; **[y-polarization] (b)** diameter −10%, −5%, +10% and +5% of ***d***_***1***_; **[x-polarization] (c)** diameter −10%, −5%, +10% and +5% of ***d***_***c***_; **[y-polarization] (d)** diameter −10%, −5%, +10% and +5% of ***d***_***c***_.

The suggested sensor's hexagonal structure may be effectively constructed using the widely popular stack-and-draw fabrication process [43]. Practically, fabricating the sensor with the precise design specifications is rather complicated. As a result, during manufacturing, deviations of up to ±1% or ±2% from the optimized values are considered. Consequently, it is important to verify the fabrication tolerance (FT) study. Fig. 15 (a), (b), (c), and (d) demonstrated fluctuation in CL was extremely small and had no effect on sensor performance, and it is insignificant for diameter dimension *d*_*1*_ & *d*_*c*_ changes of ±5% and ±10% as shown in Table 11.

**Table 11:** Fabrication Tolerance for *d*_*1*_, *d*_*c*_ & *p*.

Parameters	Tolerance (±5–10%)	Loss (x-pol) dB/cm	Loss (y-pol) dB/cm	Resonance Wavelength (μm)
d_1_	−10%	1.164	1.083	0.6
d_1_	−5%	0.816	0.773	0.6
d_1_	optimum	0.557	0.538	0.6
d_1_	+5%	0.369	0.369	0.6
d_1_	+10%	0.236	0.237	0.6
d_c_	−10%	0.633	0.617	0.6
d_c_	−5%	0.595	0.577	0.6
d_c_	optimum	0.557	0.538	0.6
d_c_	+5%	0.520	0.500	0.6
d_c_	+10%	0.485	0.463	0.6
p	+10%	0.747	0.690	0.6
p	+5%	0.655	0.620	0.6
p	optimum	0.557	0.538	0.6

### 3.9 Fabrication procedure

**Fig. 16:**
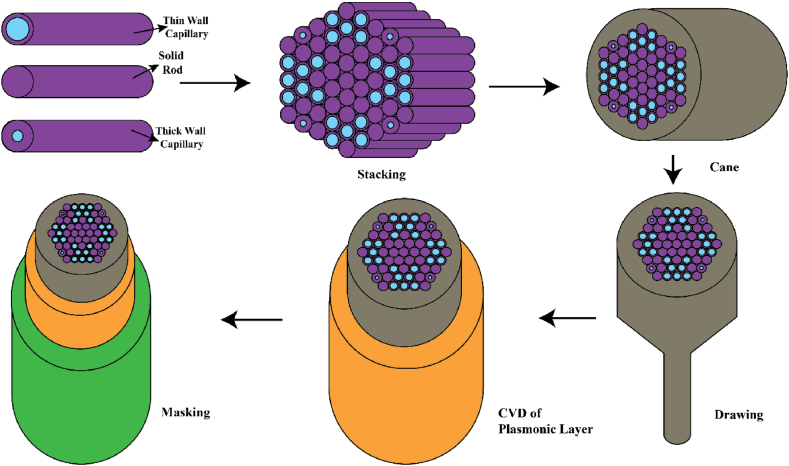
Step by step fabrication procedure of proposed sensor.

Our proposed design will be composed of circular air holes only which ensure the practical realization of the sensor would be simple and straightforward. The fabrication process of the PCF can be divided into a few stages, including structure design, air hole drilling by Stack & Draw method, preform stacking, preform fusing and final drawing. Liangliang Lv et al. [77] suggested a novel PCF structure with distributed micro holes on its core with 3 different size air hole diameters of dimensions d_1_ = 2.0 μm, d_2_ = 2.4 μm and d_3_ = 0.2 μm respectively. The PCF structure has been correctly found to be sensitive for the temperature sensing purpose with detection range of 23 °C when the diameter d_3_ is 0.20 μm. As for the pitch size to be 1 μm, the fabrication process can be done following the stack-and-draw method mentioned by MR Islam et al. [48] where the optimized pitch size was also considered as 1 μm. Also, pitch size of 0.65 μm and air hole diameter of 0.11 μm has been fabricated adopting the stack-and-draw method by Wiederhecker et al. [78]. The proposed PCF construction technique is depicted in Fig. 16. Popularly used stack & draw approach can be performed to assemble the sensor's core & cladding portion [67]. Initially, two types of capillaries (thick wall & thin wall) are produced. The capillaries need to be produced 100 times larger than the specified size. These capillaries, together with solid rod structures, will be organized in accordance with the proposed sensor's air hole arrangement. The transitional preform-cane will then be drawn when the proposed measurements are met. Next, the layer of TiO_2_ can be applied around the fiber surface using chemical vapor deposition technique [38]. Then according to design preference plasmonic layer (Au or AZO) will be deposited above TiO_2_ layer using same method. For sensing layer, two pumps will be needed one for letting the analyte enter; another for evacuating the sensing layer [24]. Employing a technique known as splicing, it would be possible to properly couple the sensor with SMF-28. Splicing method that attaches the SMF and the PCF by inserting an etched SMF tip into the PCF has been reported to have an 84.5% coupling efficiency [79]. Using the Vytran FFS-2000 splicer and the filament fusion method, the SMF and PCF can be aligned using manual-mode translational and rotational alignment [80]. In addition, numerous high-efficiency SMF-PCF couplers have been reported by researchers [81,82]; these couplers can also be utilized for our purposes. Therefore, using existing fabrication techniques our proposed sensor can be fabricated by following these steps.

## 4 Conclusion

A multifunctional SPR-PCF sensor capable of RI sensing, temperature sensing as well as verifying it by DS-DPSS is proposed in this manuscript. Gold and AZO are both used as the plasmonic material exhibiting different result and different sensing capabilities. Maximum AS for gold was found as 5269 RIU^−1^ and 5336 RIU^−1^ for x and y polarization respectively and WS was 36,500 nm/RIU for x polarization and 40500nm/RIU for y polarization. AZO shows AS of 3593 RIU^−1^ and 3763 RIU^−1^ for x and y polarization respectively and WS of 8500 nm/RIU and 9100 nm/RIU for x and y respectively. The sensor offered a stunning RI detection range of 1.19–1.42 when AZO was used whereas it was 1.32–1.43 for Gold. A sensor resolution, having Gold as the plasmonic material, of 46.4 × 10^−6^ RIU for x-pol and 46.3 × 10^−6^ RIU for y pol were found for amplitude and 100 × 10^−6^ RIU was found for wavelength for both x and y-pol. Incorporating AZO, resulted in Amplitude Resolution of 16.1 × 10^−6^ RIU for x-pol and 15.5 × 10^−6^ for y-pol and Wavelength Resolution of 200 × 10^−6^ was found for both x and y-pol. Temperature sensitivity obtained were 1.05 nm/^°^C & 1 nm/^°^C having a resolution of 0.095 °C and 0.1 °C respectively for x and y-pol. Furthermore, for double step verification that was proposed as a novel feature in this paper, maximum DPSS was 0.35 nm/^°^C for x-pol and 0.3 nm/^°^C for y-pol. The tolerance exhibited by this sensor is exquisite which shows up to ±10% change in the geometric parameters so it can be easily realized using Stack & Draw process. This sensor along with its different sensing abilities can be a major contributor to advanced analyte sensing using SPR and the DS-DPSS can prove as a major breakthrough in opening a whole other world of temperature sensing.

## Author contribution statement

Mohammad Rakibul Islam; Md. Moinul Islam Khan; Ahmad Jarif Yeasir; Jannat Ara Mim; Tajuddin Ahmed Nahid: Conceived and designed the experiments; Performed the experiments; Analyzed and interpreted the data; Contributed reagents, materials, analysis tools or data; Wrote the paper.

Fariha Mehjabin; Jubair Alam Chowdhury; Mohibul Islam: Performed the experiments; Contributed reagents, materials, analysis tools or data.

## Funding statement

This research received no specific grant from any funding agency in the public, commercial, or not-for-profit sectors.

## Data availability statement

No data was used for the research described in the article.

## Declaration of competing interest

The authors declare that they have no known competing financial interests or personal relationships that could have appeared to influence the work reported in this paper.

## References

[1] Hasan M.R. (2018). Spiral photonic crystal fiber-based dual-polarized surface plasmon resonance biosensor. IEEE Sensor. J..

[2] Yan X., Li B., Cheng T., Li S. (2018). Analysis of high sensitivity photonic crystal fiber sensor based on surface plasmon resonance of refractive indexes of liquids. Sensors.

[3] Chen X., Xia L., Li C. (2018). Surface plasmon resonance sensor based on a novel D-shaped photonic crystal fiber for low refractive index detection. IEEE Photon. J..

[4] Rifat A.A., Hasan M.R., Ahmed R., Butt H. (2017). Photonic crystal fiber-based plasmonic biosensor with external sensing approach (erratum). J. Nanophotonics.

[5] Ibrahim J. (2019). Surface plasmon resonance based temperature sensors in liquid environment. Sensors.

[6] Zhou X. (2020). High-sensitivity SPR temperature sensor based on hollow-core fiber. IEEE Trans. Instrum. Meas..

[7] Yang W. (2021). The polydimethylsiloxane coated fiber optic for all fiber temperature sensing based on the multithin-multifiber structure. IEEE Sensor. J..

[8] Wang Y., Huang Q., Zhu W., Yang M., Lewis E. (2019). Novel optical fiber SPR temperature sensor based on MMF-PCF-MMF structure and gold-PDMS film: erratum. Opt Express.

[9] Wang F., Sun Z., Sun T., Liu C., Chu P.K., Bao L. (2018). Highly sensitive PCF-SPR biosensor for hyperthermia temperature monitoring. J. Opt..

[10] Iu C.H.A.O.L. (2018). Symmetrical dual D-shape photonic crystal fibers for surface plasmon r. Opt Express.

[11] Maharana P.K., Jha R., Palei S. (Jan. 2014). Sensitivity enhancement by air mediated graphene multilayer based surface plasmon resonance biosensor for near infrared. Sensor. Actuator. B Chem..

[12] Hasan M.R., Akter S., Ahmed K., Abbott D. (2018). Plasmonic refractive index sensor employing niobium nanofilm on photonic crystal fiber. IEEE Photon. Technol. Lett..

[13] Islam M.S. (2018).

[14] Islam M.R., Iftekher A.N.M., Mou F.A., Rahman M.M., Bhuiyan M.I.H. (2021). Design of a Topas-based ultrahigh-sensitive PCF biosensor for blood component detection. Appl. Phys. Mater. Sci. Process.

[15] Islam M.A., Islam M.R., Al Naser A.M., Anzum F., Jaba F.Z. (2021). Square structured photonic crystal fiber based THz sensor design for human body protein detection. J. Comput. Electron..

[16] Rakibul Islam M., Iftekher A.N.M., Rakibul Hasan K., Nayen M.J., Bin Islam S. (Apr. 2020). Dual-polarized highly sensitive surface-plasmon-resonance-based chemical and biomolecular sensor. Appl. Opt..

[17] Aminul Islam M., Rakibul Islam M., Moinul Islam Khan M., Chowdhury J.A., Mehjabin F., Islam M. (2020). Highly birefringent slotted core photonic crystal fiber for THz wave propagation. Phys. Wave Phenom..

[18] Moshiur Rahman M., Akter Mou F., Imamul Hassan Bhuiyan M., Rakibul Islam M. (2020). Design and characterization of a circular sectored core cladding structured photonic crystal fiber with ultra-low EML and flattened dispersion in the THz regime. Opt. Fiber Technol..

[19] Sultana J., Islam M.R., Faisal M., Abu Talha K.M., Islam M.S. (Jan. 2019). Design and analysis of a Zeonex based diamond-shaped core kagome lattice photonic crystal fiber for T-ray wave transmission. Opt. Fiber Technol..

[20] Islam M.R., Kabir M.F., Talha K.M.A., Arefin M.S. (2020). Highly birefringent honeycomb cladding terahertz fiber for polarization-maintaining applications. Opt. Eng..

[21] An G., Li S., Yan X., Zhang X., Yuan Z., Zhang Y. (2016). High-sensitivity and tunable refractive index sensor based on dual-core photonic crystal fiber. J. Opt. Soc. Am. B.

[22] Qazi H.H., Bin Mohammad A.B., Akram M. (2012). Recent progress in optical chemical sensors. Sensors.

[23] Upadhyay A., Singh S., Prajapati Y.K., Tripathi R. (2020). Numerical analysis of large negative dispersion and highly birefringent photonic crystal fiber. Optik.

[24] Islam M.S. (2018). Dual-polarized highly sensitive plasmonic sensor in the visible to near-IR spectrum. Opt Express.

[25] Li D., Zhang W., Liu H., Hu J., Zhou G. (2017). High sensitivity refractive index sensor based on multicoating photonic crystal fiber with surface plasmon resonance at near-infrared wavelength. IEEE Photon. J..

[26] Momota M.R., Hasan M.R. (Feb. 2018). Hollow-core silver coated photonic crystal fiber plasmonic sensor. Opt. Mater..

[27] West P.R., Ishii S., Naik G.V., Emani N.K., Shalaev V.M., Boltasseva A. (2010). Searching for better plasmonic materials. Laser Photon. Rev..

[28] Khaleque A., Mironov E.G., Hattori H.T. (2015). Analysis of the properties of a dual-core plasmonic photonic crystal fiber polarization splitter. Appl. Phys. B Laser Opt..

[29] Khaleque A., Hattori H.T. (2015). Polarizer based upon a plasmonic resonant thin layer on a squeezed photonic crystal fiber. Appl. Opt..

[30] Qin W., Li S., Yao Y., Xin X., Xue J. (2014). Analyte-filled core self-calibration microstructured optical fiber based plasmonic sensor for detecting high refractive index aqueous analyte. Opt Laser. Eng..

[31] Gangwar R.K., Singh V.K. (2017). Highly sensitive surface plasmon resonance based D-shaped photonic crystal fiber refractive index sensor. Plasmonics.

[32] Danlard I., Akowuah E.K. (2021). Design and theoretical analysis of a dual-polarized quasi D-shaped plasmonic PCF microsensor for back-to-back measurement of refractive index and temperature. IEEE Sensor. J..

[33] Mumtaz F. (Dec. 2022). MXene (Ti3C2Tx) coated highly-sensitive D-shaped photonic crystal fiber based SPR-biosensor. Photon. Nanostruct: Fundam. Appl..

[34] Shakya A.K., Ramola A., Singh S., Van V. (2022). Design of an ultra-sensitive bimetallic anisotropic PCF SPR biosensor for liquid analytes sensing. Opt Express.

[35] Islam M.R. (2022).

[36] Wang J.-K., Ying Y., Gao Z.-J., Cheng S.-Y., Si G.-Y. (2022). Surface plasmon resonance (SPR) based temperature and magnetic field sensor in a dual-core D-shaped photonic crystal fiber (PCF). Instrum. Sci. Technol..

[37] Mumtaz F., Zhang B., Roman M., Abbas L.G., Ashraf M.A., Dai Y. (Feb. 2023). Computational study: windmill-shaped multi-channel SPR sensor for simultaneous detection of multi-analyte. Measurement.

[38] Al Mahfuz M., Hasan M.R., Momota M.R., Masud A., Akter S. (2019). Asymmetrical photonic crystal fiber based plasmonic sensor using the lower birefringence peak method. OSA Contin.

[39] Haider F., Aoni R.A., Ahmed R., Islam M.S., Miroshnichenko A.E. (2019). Propagation controlled photonic crystal fiber-based plasmonic sensor via scaled-down approach. IEEE Sensor. J..

[40] Islam Mohammad Rakibul (2021). Highly birefringent gold-coated SPR sensor with extremely enhanced amplitude and wavelength sensitivity. Eur. Phys. J. A.

[41] Islam M.R., Iftekher A.N.M., Noor F., Khan M.R.H., Reza M.T., Nishat M.M. (2022). AZO-coated plasmonic PCF nanosensor for blood constituent detection in near-infrared and visible spectrum. Appl. Phys. Mater. Sci. Process.

[42] Islam M.S., Islam M.R., Sultana J., Dinovitser A., Ng B.W.-H., Abbott D. (2019). Exposed-core localized surface plasmon resonance biosensor. J. Opt. Soc. Am. B.

[43] Azman M.F., Mahdiraji G.A., Wong W.R., Aoni R.A., Adikan F.R.M. (Mar. 2019). Design and fabrication of copper-filled photonic crystal fiber based polarization filters. Appl. Opt..

[44] Haque E., Hossain M.A., Ahmed F., Namihira Y. (2018). Surface plasmon resonance sensor based on modified D-shaped photonic crystal fiber for wider range of refractive index detection. IEEE Sensor. J..

[45] Islam M.R. (2021). Design and numerical analysis of a gold-coated photonic crystal fiber based refractive index sensor. Opt. Quant. Electron..

[46] Islam M.R. (2023). Design of a dual spider-shaped surface plasmon resonance-based refractometric sensor with high amplitude sensitivity. IET Optoelectron..

[47] Islam S., Cordeiro C.M.B., Dorraki M., Dinovitser A. (2019). A hi-Bi ultra-sensitive surface plasmon. IEEE Access.

[48] Islam M.R. (2022). Design and analysis of a QC-SPR-PCF sensor for multipurpose sensing with supremely high FOM. Appl. Nanosci..

[49] Islam M.R. (2020). Design and analysis of birefringent SPR based PCF biosensor with ultra-high sensitivity and low loss. Optik.

[50] Thenmozhi H., Mani Rajan M.S., Ahmed K. (2019). D-shaped PCF sensor based on SPR for the detection of carcinogenic agents in food and cosmetics. Optik.

[51] Kaur V., Singh S. (2019). Design of titanium nitride coated PCF-SPR sensor for liquid sensing applications. Opt. Fiber Technol..

[52] Islam M.R. (2021). Design and analysis of a biochemical sensor based on surface plasmon resonance with ultra-high sensitivity. Plasmonics.

[53] Islam M.R. (2021). Surface plasmon resonance based highly sensitive gold coated PCF biosensor. Appl. Phys. Mater. Sci. Process.

[54] Haider F., Aoni R.A., Ahmed R., Miroshnichenko A.E. (2018). Highly amplitude-sensitive photonic-crystal-fiber-based plasmonic sensor. J. Opt. Soc. Am. B.

[55] Naik G.V., Shalaev V.M., Boltasseva A. (2013). Alternative plasmonic materials: beyond gold and silver. Adv. Mater..

[56] Lewis B.G., Paine D.C. (2000).

[57] Dixon S.C., Scanlon D.O., Carmalt C.J., Parkin I.P. (2016). N-Type doped transparent conducting binary oxides: an overview. J. Mater. Chem. C.

[58] Haque E., Mahmuda S., Hossain M.A., Hai N.H., Namihira Y., Ahmed F. (2019). Highly sensitive dual-core PCF based plasmonic refractive index sensor for low refractive index detection. IEEE Photon. J..

[59] Yang Z., Xia L., Li C., Chen X., Liu D. (2019). A surface plasmon resonance sensor based on concave-shaped photonic crystal fiber for low refractive index detection. Opt Commun..

[60] Dash J.N., Das R., Jha R. (2018). AZO coated microchannel incorporated PCF-based SPR sensor: a numerical analysis. IEEE Photon. Technol. Lett..

[61] Rakibul Islam M., Iftekher A.N.M., Anzum M.S., Rahman M., Siraz S. (2022). LSPR based double peak double plasmonic layered bent core PCF-SPR sensor for ultra-broadband dual peak sensing. IEEE Sensor. J..

[62] Yu Y. (2010). Some features of the photonic crystal fiber temperature sensor with liquid ethanol filling. Opt Express.

[63] Xue Z., Cheng Z., Xu J., Xiang Q., Wang X., Xu J. (2017). Controllable evolution of dual defect zni and VO associate-rich ZnO nanodishes with (0001) exposed facet and its multiple sensitization effect for ethanol detection. ACS Appl. Mater. Interfaces.

[64] Li S.M. (Oct. 2017). Acetone sensing of ZnO nanosheets synthesized using room-temperature precipitation. Sensor. Actuator. B Chem..

[65] Hadiyan M., Salehi A., Koohi-Saadi A. (2019). Sub-ppm acetone gas sensing properties of free-standing ZnO nanorods. J. Electroceram..

[66] Sankar Ganesh R. (Oct. 2017). Low temperature ammonia gas sensor based on Mn-doped ZnO nanoparticle decorated microspheres. J. Alloys Compd..

[67] Rezaie M.N., Manavizadeh N., Nayeri F.D., Bidgoli M.M., Nadimi E., Boroumand F.A. (2018). Effect of seed layers on low-temperature, chemical bath deposited ZnO nanorods-based near UV-OLED performance. Ceram. Int..

[68] Gamal Y., Younis B.M., Hegazy S.F., Badr Y., Hameed M.F.O., Obayya S.S.A. (2022). Highly sensitive multi-functional plasmonic biosensor based on dual core photonic crystal fiber. IEEE Sensor. J..

[69] Rakibul Islam M., Khan M.M.I., Mehjabin F., Alam Chowdhury J., Islam M. (2020). Design of a fabrication friendly & highly sensitive surface plasmon resonance-based photonic crystal fiber biosensor. Results Phys..

[70] Abbasi M., Soroosh M., Namjoo E. (2018). Polarization-insensitive temperature sensor based on liquid filled photonic crystal fiber. Optik.

[71] Anjum N.M., Mumtaz F., Ashraf M.A. (May 2023). Design and analysis of Gold-nanowires based multi-channel SPR sensor. Results Opt.

[72] Mo X., Lv J., Liu Q., Jiang X., Si G. (2021). A magnetic field SPR sensor based on temperature self-reference. Sensors.

[73] Liu S., Cao S., Zhang Z., Wang Y., Liao C., Wang Y. (2019). Temperature sensor based on side-polished fiber SPR device coated with polymer. Sensors.

[74] Islam M.R. (2022). Trigonal cluster-based ultra-sensitive surface plasmon resonance sensor for multipurpose sensing. Sens. Bio-Sensing Res..

[75] Hasan M.K., Rahman M.M., Anower M.S., Rana M.M., Paul A.K., Chakrabatri K. (2020). 2020 IEEE Reg. 10 Symp. TENSYMP 2020.

[76] Mumtaz F., Dai Y., Ashraf M.A. (2020). Inter-cross de-modulated refractive index and temperature sensor by an etched multi-core fiber of a MZI structure. J. Lightwave Technol..

[77] Lv L., Liu Q., Xue P. (Sep. 2020). The sensing characteristics of microstructure-core photonic crystal fiber filled with liquid based on Sagnac interferometer. Results Phys..

[78] Wiederhecker G.S. (2007). Field enhancement within an optical fibre with a subwavelength air core. Nat. Photonics.

[79] Fan D. (2017). Extremely high-efficiency coupling method for hollow-core photonic crystal fiber. IEEE Photon. J..

[80] Murawski M., Jaroszewicz L.R., Stasiewicz K. (2009). A photonic crystal fiber splice with a standard single mode fiber. Photonics Lett. Pol..

[81] Wei H., Zhu Y., Krishnaswamy S. (2015). Numerical analysis of waveguide coupling between photonic crystal fiber and single-mode fiber. IEEE Photon. Technol. Lett..

[82] Yokota Hirohisa, Yashima Hirotomo, Imai Yoh, Sasaki Yutaka (2016). Coupling characteristics of fused optical fiber coupler formed with single-mode fiber and photonic crystal fiber having air hole collapsed taper. Adv. Optoelectron..

